# 7-[(7*S*)-7-Aza­niumyl-5-aza­spiro­[2.4]hept-5-yl]-8-chloro-6-fluoro-1-[(1*S*,2*R*)-2-fluoro­cyclo­prop­yl]-4-oxo-1,4-dihydroquinoline-3-carboxyl­ate methanol monosolvate

**DOI:** 10.1107/S160053681203632X

**Published:** 2012-08-25

**Authors:** Wen-jie Xu, Xue-hui Qiu, Huai-jie Hua, Song-de Tan, Hai-yan Ding

**Affiliations:** a Shenzhen Salubris Pharmaceuticals Co. Ltd, Guangdong Shenzhen 361021, People’s Republic of China; b Guangzhou Institutes of Biomedicine and Health, Chinese Academy of Sciences, Guanzhou, Guangdong 510530, People’s Republic of China

## Abstract

Sitafloxacin is a newly developed fluoro­quinolone anti­bacterial drug. The crystal studied, C_19_H_18_ClF_2_N_3_O_3_·CH_3_OH, consists of one mol­ecule of sitafloxacin and one methanol solvent mol­ecule. The mol­ecule of sitafloxacin is a zwitterion with a protonated primary amine group and a deprotonated carboxylate group. The cyclopropane ring and the CO_2_ group make dihedral angles of 79.5 (3) and 35.4 (4)°, respectively, with the fused ring system. The supra­molecular structure is defined by N—H⋯O and O—H⋯O hydrogen bonds.

## Related literature

For the synthesis, applications and pseudopolymorphic structure of the title compound, see: Yamazaki *et al.* (1998 ▶), Suzuki *et al.* (2000 ▶) and Suzuki *et al.* (2010 ▶), respectively. 
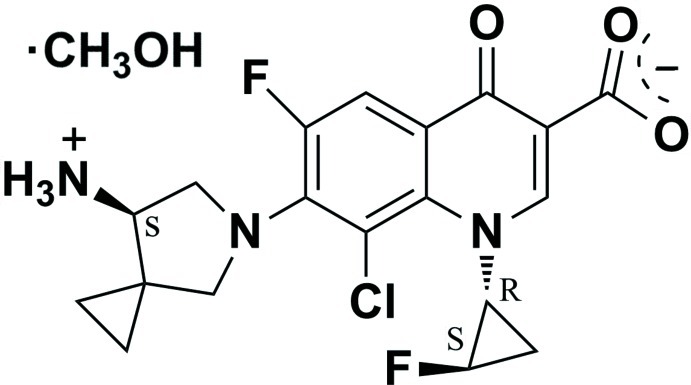


## Experimental

### 

#### Crystal data

C_19_H_18_ClF_2_N_3_O_3_·CH_4_O
*M* *_r_* = 441.86
Monoclinic, 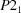

*a* = 8.7455 (3) Å
*b* = 8.2968 (3) Å
*c* = 14.0638 (4) Å
β = 104.474 (3)°
*V* = 988.07 (5) Å^3^
*Z* = 2
Cu *K*α radiation
μ = 2.18 mm^−1^
*T* = 293 K
0.2 × 0.1 × 0.05 mm

#### Data collection

Agilent Xcalibur Sapphire3 Gemini ultra diffractometer
Absorption correction: multi-scan (*CrysAlis PRO*; Agilent, 2011 ▶) *T*_min_ = 0.990, *T*_max_ = 1.000
5115 measured reflections
2745 independent reflections
2537 reflections with *I* > 2σ(*I*)
*R*_int_ = 0.033

#### Refinement

*R*[*F* ^2^ > 2σ(*F* ^2^)] = 0.038
*wR*(*F* ^2^) = 0.100
*S* = 1.08
2745 reflections
273 parameters
1 restraint
H-atom parameters constrained
Δρ_max_ = 0.21 e Å^−3^
Δρ_min_ = −0.21 e Å^−3^
Absolute structure: Flack, (1983 ▶), 856 Friedel pairs
Flack parameter: −0.024 (19)

### 

Data collection: *CrysAlis PRO* (Agilent, 2011 ▶); cell refinement: *CrysAlis PRO*; data reduction: *CrysAlis PRO*; program(s) used to solve structure: *OLEX2* (Dolomanov *et al.*, 2009 ▶); program(s) used to refine structure: *OLEX2*; molecular graphics: *OLEX2*; software used to prepare material for publication: *OLEX2*.

## Supplementary Material

Crystal structure: contains datablock(s) I, global. DOI: 10.1107/S160053681203632X/go2064sup1.cif

Structure factors: contains datablock(s) I. DOI: 10.1107/S160053681203632X/go2064Isup2.hkl

Supplementary material file. DOI: 10.1107/S160053681203632X/go2064Isup3.cml

Additional supplementary materials:  crystallographic information; 3D view; checkCIF report

## Figures and Tables

**Table 1:** Hydrogen-bond geometry (Å, °)

*D*—H⋯*A*	*D*—H	H⋯*A*	*D*⋯*A*	*D*—H⋯*A*
N3—H3*A*⋯O3^i^	0.89	2.07	2.929 (3)	161
N3—H3*A*⋯O2^i^	0.89	2.43	3.022 (3)	124
O4—H4*O*⋯O3^ii^	0.82	1.84	2.654 (4)	173
N3—H3*C*⋯O2^iii^	0.89	1.82	2.675 (4)	159
N3—H3*B*⋯O4	0.89	1.91	2.788 (4)	171

Symmetry codes: (i) 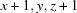; (ii) 
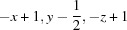
; (iii) 
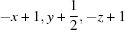
.

**Fig. 1.**
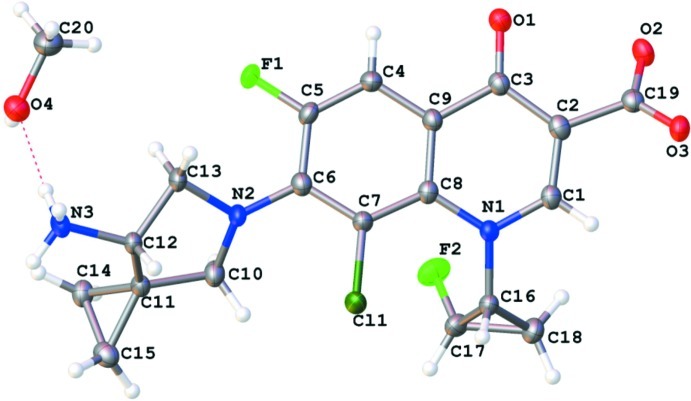
The molecular structure of the title compound showing the atom numbering scheme. Hydrogen bonds are shown as dashed lines and displacement ellipsoids are drawn at the 50% probability level.

## supplementary crystallographic information

### Comment

Three hydrate forms (hemihydrate, monohydrade and sesqiuhydrate) and three anhydrate forms (alpha,beta and gamma forms) of sitafloxacin crystals have been found so far. The polymorphic and pseudopolymorphic crystals show different physicochemical properties (Yamazaki *et al.*, 1998, Suzuki *et al.*, 2000). The crystal structures of the beta form, the monohydrate and the sesquihydrate have been already reported (Yamazaki *et al.*, 1998, Suzuki *et al.*, 2000). Preparation of the title compound has been reported earlier (Suzuki *et al.*, 2010). In this paper, we report the X-ray crystal structure of the title compound. All donor H atoms are involved in the hydrogen bonding as well as all acceptor O atoms with exception made for O1, Table 1.

### Experimental

The preparation of the titled compound was made following a similar procedure described earlier (Suzuki *et al.*, 2010). The 7-[(7*S*)-7-Boc-NH-5-azaspiro[2.4]hept-5-yl]-6-fluoro-1-[(1*S*,2*R*)-2- fluorocyclopropyl]-1,4-dihydro-4-oxo-3-quinolinecarboxylic acid reacted with thionyl chloride in dichloromethane for 8 h. The yield was 78%. The Boc functional group was removed with trifluoroacetic acid,. The resulting solution was concentrated, washed giving the colorless crystals of the title compound, yield: 68%. 0.2g of this material was dissolved in 20 ml of methanol at 323K. Slow evaporation gave needle-like crystals suitable for X-ray analysis.

### Refinement

The asymmetric unit was selected so that the molecule and the methanol solvent form a hydrgen bonded unit. All hydrogen atoms were located. The carbon-bonded H atoms were placed in idealized positions C—H = 0.93–0.98, *U*_iso_(H) = 1.2–1.5Ueq(C) and were included in the refinement in the riding model approximation. The H atom of the methanol OH group were located in a difference Fourier map and then allowed to ride on his parent O atom, with O—H = 0.82Å and *U*_iso_(H) = 1.5Ueq(O). The three H atoms of the idealized NH3+ group were created by an AFIX 137 instruction N—H = 0.89Å, *U*_iso_(H) = 1.5Ueq(N).

The positions of these H atoms were checked on a final difference map.

### Crystal data

**Table:**

C_19_H_18_ClF_2_N_3_O_3_·CH_4_O	*F*(000) = 460
*M**_r_* = 441.86	*D*_x_ = 1.485 Mg m^−^^3^
Monoclinic, *P*2_1_	Cu *K*α radiation, λ = 1.54184 Å
*a* = 8.7455 (3) Å	θ = 3.3–66.9°
*b* = 8.2968 (3) Å	µ = 2.18 mm^−^^1^
*c* = 14.0638 (4) Å	*T* = 293 K
β = 104.474 (3)°	Block, colourless
*V* = 988.07 (5) Å^3^	0.2 × 0.1 × 0.05 mm
*Z* = 2	

### Data collection

**Table:**

Agilent Xcalibur Sapphire3 Gemini ultra diffractometer	2745 independent reflections
Radiation source: fine-focus sealed tube	2537 reflections with *I* > 2σ(*I*)
Graphite monochromator	*R*_int_ = 0.033
ω scans	θ_max_ = 66.9°, θ_min_ = 3.3°
Absorption correction: multi-scan (*CrysAlis PRO*; Agilent, 2011)	*h* = −9→10
*T*_min_ = 0.990, *T*_max_ = 1.000	*k* = −9→9
5115 measured reflections	*l* = −15→16

### Refinement

**Table:**

Refinement on *F*^2^	Secondary atom site location: difference Fourier map
Least-squares matrix: full	Hydrogen site location: inferred from neighbouring sites
*R*[*F*^2^ > 2σ(*F*^2^)] = 0.038	H-atom parameters constrained
*wR*(*F*^2^) = 0.100	*w* = 1/[σ^2^(*F*_o_^2^) + (0.0582*P*)^2^ + 0.0956*P*] where *P* = (*F*_o_^2^ + 2*F*_c_^2^)/3
*S* = 1.08	(Δ/σ)_max_ < 0.001
2745 reflections	Δρ_max_ = 0.21 e Å^−^^3^
273 parameters	Δρ_min_ = −0.21 e Å^−^^3^
1 restraint	Absolute structure: Flack, (1983), 856 Friedel pairs
Primary atom site location: structure-invariant direct methods	Flack parameter: −0.024 (19)

### Special details

**Table:**

Geometry. All e.s.d.'s (except the e.s.d. in the dihedral angle between two l.s. planes) are estimated using the full covariance matrix. The cell e.s.d.'s are taken into account individually in the estimation of e.s.d.'s in distances, angles and torsion angles; correlations between e.s.d.'s in cell parameters are only used when they are defined by crystal symmetry. An approximate (isotropic) treatment of cell e.s.d.'s is used for estimating e.s.d.'s involving l.s. planes.
Refinement. Refinement of *F*^2^ against ALL reflections. The weighted *R*-factor *wR* and goodness of fit *S* are based on *F*^2^, conventional *R*-factors *R* are based on *F*, with *F* set to zero for negative *F*^2^. The threshold expression of *F*^2^ > σ(*F*^2^) is used only for calculating *R*-factors(gt) *etc*. and is not relevant to the choice of reflections for refinement. *R*-factors based on *F*^2^ are statistically about twice as large as those based on *F*, and *R*- factors based on ALL data will be even larger.

### Fractional atomic coordinates and isotropic or equivalent isotropic displacement parameters (Å^2^)

**Table:**

	*x*	*y*	*z*	*U*_iso_*/*U*_eq_	
Cl1	0.85678 (8)	0.70137 (11)	0.46653 (5)	0.02508 (19)	
F1	0.4439 (2)	0.5006 (3)	0.63177 (12)	0.0273 (5)	
F2	0.7157 (2)	0.3005 (3)	0.33304 (16)	0.0357 (5)	
N1	0.5964 (3)	0.6077 (3)	0.27925 (18)	0.0203 (6)	
N2	0.7559 (3)	0.5849 (4)	0.63790 (17)	0.0230 (6)	
N3	0.9156 (3)	0.6914 (4)	0.89555 (17)	0.0243 (6)	
H3A	1.0152	0.6728	0.9281	0.036*	
H3B	0.8519	0.6240	0.9165	0.036*	
H3C	0.8899	0.7925	0.9060	0.036*	
O1	0.1338 (2)	0.5512 (3)	0.28669 (16)	0.0259 (5)	
O2	0.0906 (2)	0.5100 (3)	0.07641 (15)	0.0263 (5)	
O3	0.2332 (2)	0.6989 (3)	0.02739 (14)	0.0258 (5)	
C1	0.4763 (4)	0.6164 (4)	0.1961 (2)	0.0210 (7)	
H1A	0.5039	0.6322	0.1371	0.025*	
C2	0.3199 (4)	0.6040 (4)	0.1921 (2)	0.0225 (7)	
C3	0.2715 (3)	0.5742 (4)	0.2817 (2)	0.0211 (7)	
C4	0.3635 (4)	0.5465 (4)	0.4621 (2)	0.0228 (7)	
H4A	0.2589	0.5322	0.4642	0.027*	
C5	0.4807 (4)	0.5417 (4)	0.5467 (2)	0.0209 (7)	
C6	0.6391 (4)	0.5778 (4)	0.5504 (2)	0.0225 (7)	
C7	0.6742 (3)	0.6140 (4)	0.4608 (2)	0.0213 (7)	
C8	0.5596 (4)	0.5974 (4)	0.3706 (2)	0.0200 (7)	
C9	0.4007 (3)	0.5730 (4)	0.3720 (2)	0.0205 (7)	
C10	0.8951 (4)	0.4780 (5)	0.6564 (2)	0.0253 (8)	
H10A	0.9615	0.5022	0.6124	0.030*	
H10B	0.8639	0.3656	0.6490	0.030*	
C11	0.9791 (3)	0.5169 (4)	0.7627 (2)	0.0208 (7)	
C12	0.8986 (3)	0.6661 (4)	0.7881 (2)	0.0202 (7)	
H12A	0.9402	0.7607	0.7612	0.024*	
C13	0.7270 (4)	0.6385 (4)	0.7308 (2)	0.0232 (7)	
H13A	0.6757	0.5561	0.7607	0.028*	
H13B	0.6655	0.7371	0.7233	0.028*	
C14	1.0352 (4)	0.3823 (4)	0.8350 (2)	0.0243 (7)	
H14A	1.0215	0.2725	0.8107	0.029*	
H14B	1.0249	0.3966	0.9016	0.029*	
C15	1.1523 (4)	0.4884 (5)	0.8009 (2)	0.0275 (8)	
H15A	1.2122	0.5658	0.8472	0.033*	
H15B	1.2088	0.4418	0.7564	0.033*	
C16	0.7537 (3)	0.5663 (4)	0.2680 (2)	0.0207 (7)	
H16A	0.8311	0.6541	0.2790	0.025*	
C17	0.8167 (4)	0.4026 (5)	0.3007 (2)	0.0255 (8)	
H17A	0.9297	0.3938	0.3324	0.031*	
C18	0.7710 (4)	0.4402 (5)	0.1939 (2)	0.0274 (8)	
H18A	0.6755	0.3911	0.1543	0.033*	
H18B	0.8549	0.4544	0.1607	0.033*	
C19	0.2061 (4)	0.6049 (4)	0.0915 (2)	0.0226 (7)	
O4	0.6931 (2)	0.5098 (3)	0.96034 (16)	0.0306 (6)	
H4O	0.7168	0.4143	0.9593	0.046*	
C20	0.5279 (4)	0.5273 (6)	0.9249 (3)	0.0400 (10)	
H20A	0.4963	0.4929	0.8577	0.060*	
H20B	0.4996	0.6383	0.9293	0.060*	
H20C	0.4755	0.4625	0.9638	0.060*	

### Atomic displacement parameters (Å^2^)

**Table:**

	*U*^11^	*U*^22^	*U*^33^	*U*^12^	*U*^13^	*U*^23^
Cl1	0.0194 (3)	0.0357 (5)	0.0191 (3)	−0.0057 (3)	0.0029 (3)	−0.0007 (4)
F1	0.0257 (10)	0.0426 (13)	0.0147 (9)	−0.0042 (9)	0.0073 (7)	0.0033 (9)
F2	0.0397 (11)	0.0307 (12)	0.0426 (12)	−0.0026 (9)	0.0213 (9)	0.0067 (10)
N1	0.0178 (12)	0.0267 (17)	0.0157 (12)	0.0005 (11)	0.0029 (10)	0.0018 (12)
N2	0.0193 (13)	0.0366 (17)	0.0124 (12)	0.0030 (12)	0.0027 (10)	−0.0009 (12)
N3	0.0237 (12)	0.0302 (16)	0.0159 (12)	0.0016 (13)	−0.0004 (10)	−0.0028 (14)
O1	0.0216 (11)	0.0337 (15)	0.0212 (11)	−0.0012 (10)	0.0031 (9)	0.0013 (11)
O2	0.0255 (12)	0.0295 (14)	0.0201 (11)	−0.0029 (10)	−0.0018 (9)	−0.0025 (11)
O3	0.0270 (11)	0.0330 (14)	0.0167 (10)	0.0022 (12)	0.0040 (8)	0.0017 (12)
C1	0.0234 (15)	0.0207 (18)	0.0179 (14)	0.0015 (13)	0.0030 (12)	0.0016 (14)
C2	0.0239 (16)	0.0204 (19)	0.0212 (16)	0.0015 (13)	0.0019 (13)	0.0007 (15)
C3	0.0208 (16)	0.0220 (17)	0.0193 (15)	0.0005 (13)	0.0027 (12)	−0.0016 (14)
C4	0.0181 (15)	0.030 (2)	0.0202 (15)	0.0001 (13)	0.0051 (12)	−0.0003 (15)
C5	0.0241 (16)	0.0242 (19)	0.0159 (14)	0.0005 (13)	0.0079 (12)	−0.0003 (13)
C6	0.0219 (15)	0.0264 (19)	0.0182 (14)	0.0011 (13)	0.0032 (12)	−0.0011 (15)
C7	0.0184 (15)	0.0281 (19)	0.0173 (15)	−0.0009 (13)	0.0039 (12)	0.0014 (14)
C8	0.0223 (15)	0.0203 (18)	0.0170 (14)	0.0000 (13)	0.0043 (12)	0.0003 (14)
C9	0.0200 (15)	0.0235 (18)	0.0168 (14)	0.0009 (13)	0.0026 (12)	−0.0018 (14)
C10	0.0273 (18)	0.030 (2)	0.0175 (15)	0.0056 (14)	0.0036 (13)	0.0011 (15)
C11	0.0193 (15)	0.0259 (18)	0.0160 (14)	−0.0018 (13)	0.0024 (12)	−0.0002 (14)
C12	0.0192 (14)	0.027 (2)	0.0139 (13)	0.0003 (12)	0.0023 (11)	0.0019 (13)
C13	0.0230 (16)	0.0320 (19)	0.0136 (14)	0.0012 (14)	0.0030 (12)	−0.0001 (14)
C14	0.0294 (17)	0.0232 (18)	0.0191 (15)	0.0027 (14)	0.0042 (13)	0.0013 (14)
C15	0.0248 (18)	0.028 (2)	0.0273 (17)	0.0037 (14)	0.0030 (13)	−0.0040 (16)
C16	0.0190 (15)	0.0270 (18)	0.0161 (14)	−0.0009 (13)	0.0043 (11)	0.0002 (14)
C17	0.0239 (16)	0.031 (2)	0.0233 (16)	0.0005 (14)	0.0089 (13)	0.0045 (15)
C18	0.0276 (18)	0.035 (2)	0.0195 (16)	0.0015 (15)	0.0064 (13)	−0.0025 (16)
C19	0.0217 (16)	0.024 (2)	0.0214 (16)	0.0034 (14)	0.0046 (13)	−0.0009 (15)
O4	0.0255 (12)	0.0366 (15)	0.0283 (12)	0.0029 (11)	0.0040 (9)	0.0031 (12)
C20	0.0300 (19)	0.055 (3)	0.036 (2)	0.0090 (18)	0.0104 (16)	0.005 (2)

### Geometric parameters (Å, º)

**Table:**

Cl1—C7	1.737 (3)	C10—C11	1.526 (4)
F1—C5	1.358 (3)	C10—H10A	0.9700
F2—C17	1.381 (4)	C10—H10B	0.9700
N1—C1	1.364 (4)	C11—C15	1.494 (4)
N1—C8	1.402 (4)	C11—C14	1.507 (5)
N1—C16	1.464 (4)	C11—C12	1.510 (5)
N2—C6	1.390 (4)	C12—C13	1.533 (4)
N2—C13	1.460 (4)	C12—H12A	0.9800
N2—C10	1.476 (4)	C13—H13A	0.9700
N3—C12	1.496 (4)	C13—H13B	0.9700
N3—H3A	0.8900	C14—C15	1.516 (5)
N3—H3B	0.8900	C14—H14A	0.9700
N3—H3C	0.8900	C14—H14B	0.9700
O1—C3	1.239 (4)	C15—H15A	0.9700
O2—C19	1.256 (4)	C15—H15B	0.9700
O3—C19	1.258 (4)	C16—C17	1.494 (5)
C1—C2	1.359 (4)	C16—C18	1.511 (5)
C1—H1A	0.9300	C16—H16A	0.9800
C2—C3	1.448 (4)	C17—C18	1.488 (5)
C2—C19	1.513 (4)	C17—H17A	0.9800
C3—C9	1.474 (4)	C18—H18A	0.9700
C4—C5	1.363 (4)	C18—H18B	0.9700
C4—C9	1.402 (4)	O4—C20	1.414 (4)
C4—H4A	0.9300	O4—H4O	0.8200
C5—C6	1.406 (4)	C20—H20A	0.9600
C6—C7	1.401 (4)	C20—H20B	0.9600
C7—C8	1.414 (4)	C20—H20C	0.9600
C8—C9	1.409 (4)		
			
C1—N1—C8	118.9 (3)	N3—C12—C13	112.9 (2)
C1—N1—C16	117.6 (2)	C11—C12—C13	101.9 (3)
C8—N1—C16	121.5 (2)	N3—C12—H12A	109.0
C6—N2—C13	123.5 (3)	C11—C12—H12A	109.0
C6—N2—C10	121.4 (3)	C13—C12—H12A	109.0
C13—N2—C10	110.1 (2)	N2—C13—C12	98.7 (2)
C12—N3—H3A	109.5	N2—C13—H13A	112.0
C12—N3—H3B	109.5	C12—C13—H13A	112.0
H3A—N3—H3B	109.5	N2—C13—H13B	112.0
C12—N3—H3C	109.5	C12—C13—H13B	112.0
H3A—N3—H3C	109.5	H13A—C13—H13B	109.7
H3B—N3—H3C	109.5	C11—C14—C15	59.2 (2)
C2—C1—N1	125.7 (3)	C11—C14—H14A	117.9
C2—C1—H1A	117.2	C15—C14—H14A	117.9
N1—C1—H1A	117.2	C11—C14—H14B	117.9
C1—C2—C3	119.1 (3)	C15—C14—H14B	117.9
C1—C2—C19	117.3 (3)	H14A—C14—H14B	115.0
C3—C2—C19	123.2 (3)	C11—C15—C14	60.1 (2)
O1—C3—C2	125.2 (3)	C11—C15—H15A	117.8
O1—C3—C9	119.8 (3)	C14—C15—H15A	117.8
C2—C3—C9	115.0 (3)	C11—C15—H15B	117.8
C5—C4—C9	120.0 (3)	C14—C15—H15B	117.8
C5—C4—H4A	120.0	H15A—C15—H15B	114.9
C9—C4—H4A	120.0	N1—C16—C17	117.6 (3)
F1—C5—C4	119.0 (3)	N1—C16—C18	119.7 (3)
F1—C5—C6	118.0 (3)	C17—C16—C18	59.4 (2)
C4—C5—C6	123.0 (3)	N1—C16—H16A	116.1
N2—C6—C7	120.6 (3)	C17—C16—H16A	116.1
N2—C6—C5	122.7 (3)	C18—C16—H16A	116.1
C7—C6—C5	116.6 (3)	F2—C17—C18	115.4 (3)
C6—C7—C8	121.4 (3)	F2—C17—C16	116.3 (3)
C6—C7—Cl1	116.9 (2)	C18—C17—C16	60.9 (2)
C8—C7—Cl1	121.3 (2)	F2—C17—H17A	117.4
N1—C8—C9	118.3 (2)	C18—C17—H17A	117.4
N1—C8—C7	122.8 (3)	C16—C17—H17A	117.4
C9—C8—C7	118.8 (3)	C17—C18—C16	59.8 (2)
C4—C9—C8	119.2 (3)	C17—C18—H18A	117.8
C4—C9—C3	118.6 (3)	C16—C18—H18A	117.8
C8—C9—C3	122.2 (3)	C17—C18—H18B	117.8
N2—C10—C11	102.7 (3)	C16—C18—H18B	117.8
N2—C10—H10A	111.2	H18A—C18—H18B	114.9
C11—C10—H10A	111.2	O2—C19—O3	123.7 (3)
N2—C10—H10B	111.2	O2—C19—C2	117.9 (3)
C11—C10—H10B	111.2	O3—C19—C2	118.4 (3)
H10A—C10—H10B	109.1	C20—O4—H4O	109.5
C15—C11—C14	60.7 (2)	O4—C20—H20A	109.5
C15—C11—C12	122.4 (3)	O4—C20—H20B	109.5
C14—C11—C12	122.7 (3)	H20A—C20—H20B	109.5
C15—C11—C10	121.0 (3)	O4—C20—H20C	109.5
C14—C11—C10	120.0 (3)	H20A—C20—H20C	109.5
C12—C11—C10	105.4 (2)	H20B—C20—H20C	109.5
N3—C12—C11	114.8 (3)		
			
C8—N1—C1—C2	−5.3 (5)	O1—C3—C9—C4	−0.4 (5)
C16—N1—C1—C2	158.7 (3)	C2—C3—C9—C4	179.4 (3)
N1—C1—C2—C3	−2.0 (5)	O1—C3—C9—C8	179.6 (3)
N1—C1—C2—C19	−175.7 (3)	C2—C3—C9—C8	−0.6 (5)
C1—C2—C3—O1	−175.5 (3)	C6—N2—C10—C11	−174.7 (3)
C19—C2—C3—O1	−2.2 (6)	C13—N2—C10—C11	−18.7 (3)
C1—C2—C3—C9	4.7 (5)	N2—C10—C11—C15	−155.8 (3)
C19—C2—C3—C9	178.1 (3)	N2—C10—C11—C14	132.4 (3)
C9—C4—C5—F1	−173.7 (3)	N2—C10—C11—C12	−11.3 (3)
C9—C4—C5—C6	6.4 (6)	C15—C11—C12—N3	−58.4 (4)
C13—N2—C6—C7	144.3 (4)	C14—C11—C12—N3	15.2 (4)
C10—N2—C6—C7	−62.9 (5)	C10—C11—C12—N3	157.7 (3)
C13—N2—C6—C5	−32.8 (5)	C15—C11—C12—C13	179.1 (3)
C10—N2—C6—C5	120.0 (4)	C14—C11—C12—C13	−107.2 (3)
F1—C5—C6—N2	−5.0 (5)	C10—C11—C12—C13	35.3 (3)
C4—C5—C6—N2	174.8 (3)	C6—N2—C13—C12	−164.6 (3)
F1—C5—C6—C7	177.7 (3)	C10—N2—C13—C12	40.0 (3)
C4—C5—C6—C7	−2.4 (5)	N3—C12—C13—N2	−168.4 (3)
N2—C6—C7—C8	175.9 (3)	C11—C12—C13—N2	−44.7 (3)
C5—C6—C7—C8	−6.8 (5)	C12—C11—C14—C15	−111.7 (3)
N2—C6—C7—Cl1	−11.2 (5)	C10—C11—C14—C15	111.0 (3)
C5—C6—C7—Cl1	166.1 (3)	C12—C11—C15—C14	112.2 (3)
C1—N1—C8—C9	9.1 (5)	C10—C11—C15—C14	−109.3 (4)
C16—N1—C8—C9	−154.2 (3)	C1—N1—C16—C17	−108.0 (3)
C1—N1—C8—C7	−169.5 (3)	C8—N1—C16—C17	55.5 (4)
C16—N1—C8—C7	27.1 (5)	C1—N1—C16—C18	−39.3 (4)
C6—C7—C8—N1	−169.6 (3)	C8—N1—C16—C18	124.2 (3)
Cl1—C7—C8—N1	17.8 (5)	N1—C16—C17—F2	4.1 (4)
C6—C7—C8—C9	11.8 (5)	C18—C16—C17—F2	−105.8 (3)
Cl1—C7—C8—C9	−160.8 (3)	N1—C16—C17—C18	109.9 (3)
C5—C4—C9—C8	−1.2 (5)	F2—C17—C18—C16	107.2 (3)
C5—C4—C9—C3	178.8 (3)	N1—C16—C18—C17	−106.4 (3)
N1—C8—C9—C4	173.8 (3)	C1—C2—C19—O2	139.6 (3)
C7—C8—C9—C4	−7.6 (5)	C3—C2—C19—O2	−33.9 (5)
N1—C8—C9—C3	−6.2 (5)	C1—C2—C19—O3	−39.5 (4)
C7—C8—C9—C3	172.4 (3)	C3—C2—C19—O3	147.1 (3)

### Hydrogen-bond geometry (Å, º)

**Table:**

*D*—H···*A*	*D*—H	H···*A*	*D*···*A*	*D*—H···*A*
N3—H3*A*···O3^i^	0.89	2.07	2.929 (3)	161
N3—H3*A*···O2^i^	0.89	2.43	3.022 (3)	124
O4—H4*O*···O3^ii^	0.82	1.84	2.654 (4)	173
N3—H3*C*···O2^iii^	0.89	1.82	2.675 (4)	159
N3—H3*B*···O4	0.89	1.91	2.788 (4)	171

Symmetry codes: (i) *x*+1, *y*, *z*+1; (ii) −*x*+1, *y*−1/2, −*z*+1; (iii) −*x*+1, *y*+1/2, −*z*+1.
